# Supplementary material for: Single-cell multiomics reveals ENL mutation perturbs kidney developmental trajectory by rewiring gene regulatory landscape
Source: Nat Commun. 2024 Jul 15;15:5937. doi: 10.1038/s41467-024-50171-w (PMC11250843; doi:10.1038/s41467-024-50171-w)

**Supplementary Figures**

# Single-Cell multiomics reveals ENL mutation perturbs kidney developmental trajectory by rewiring gene regulatory landscape

Lele Song[1, 2,*], Qinglan Li[1, 2,*], Lingbo Xia[1, 2, 3], Arushi Eesha Sahay[1,2], Qi Qiu[4, 5], Yuanyuan Li[6, 7], Haitao Li[6, 7], Kotaro Sasaki[8, 9, 10], Katalin Susztak[4, 11, 12], Hao Wu[4, 5], Liling Wan[1, 2, 5, 9, #]

[1]Department of Cancer Biology, University of Pennsylvania, Philadelphia, PA, 19104, USA.

[2]Abramson Family Cancer Research Institute, Perelman School of Medicine, University of Pennsylvania, Philadelphia, PA, 19104, USA

[3]Department of the School of Engineering and Applied Science, University of Pennsylvania, Philadelphia, PA, 19104, USA

[4]Department of Genetics, University of Pennsylvania, Philadelphia, PA, 19104, USA

[5]Penn Epigenetics Institute, University of Pennsylvania, Philadelphia, PA, 19104, USA

[6]MOE Key Laboratory of Protein Sciences, Beijing Frontier Research Center for Biological Structure, School of Medicine, Tsinghua University, Beijing 100084, China

[7]Tsinghua-Peking Center for Life Sciences, Beijing 100084, China

[8]Department of Biomedical Sciences, University of Pennsylvania, School of Veterinary Medicine, Philadelphia, PA, 19104, USA

[9]Institute for Regenerative Medicine, Perelman School of Medicine, University of Pennsylvania, Philadelphia, PA, 19104, USA

[10]Department of Pathology and Laboratory Medicine, University of Pennsylvania, Perelman School of Medicine, Philadelphia, PA, 19104, USA

[11]Renal, Electrolyte, and Hypertension Division, Department of Medicine, University of Pennsylvania, Perelman School of Medicine, Philadelphia, PA, 19104, USA.

[12]Institute for Diabetes, Obesity, and Metabolism, University of Pennsylvania, Perelman School of Medicine, Philadelphia, PA, USA

[*]These authors contributed equally

[#]Correspondence should be addressed to L.W. (Liling.Wan@Pennmedicine.upenn.edu)

30 **Supplementary Figure 1**

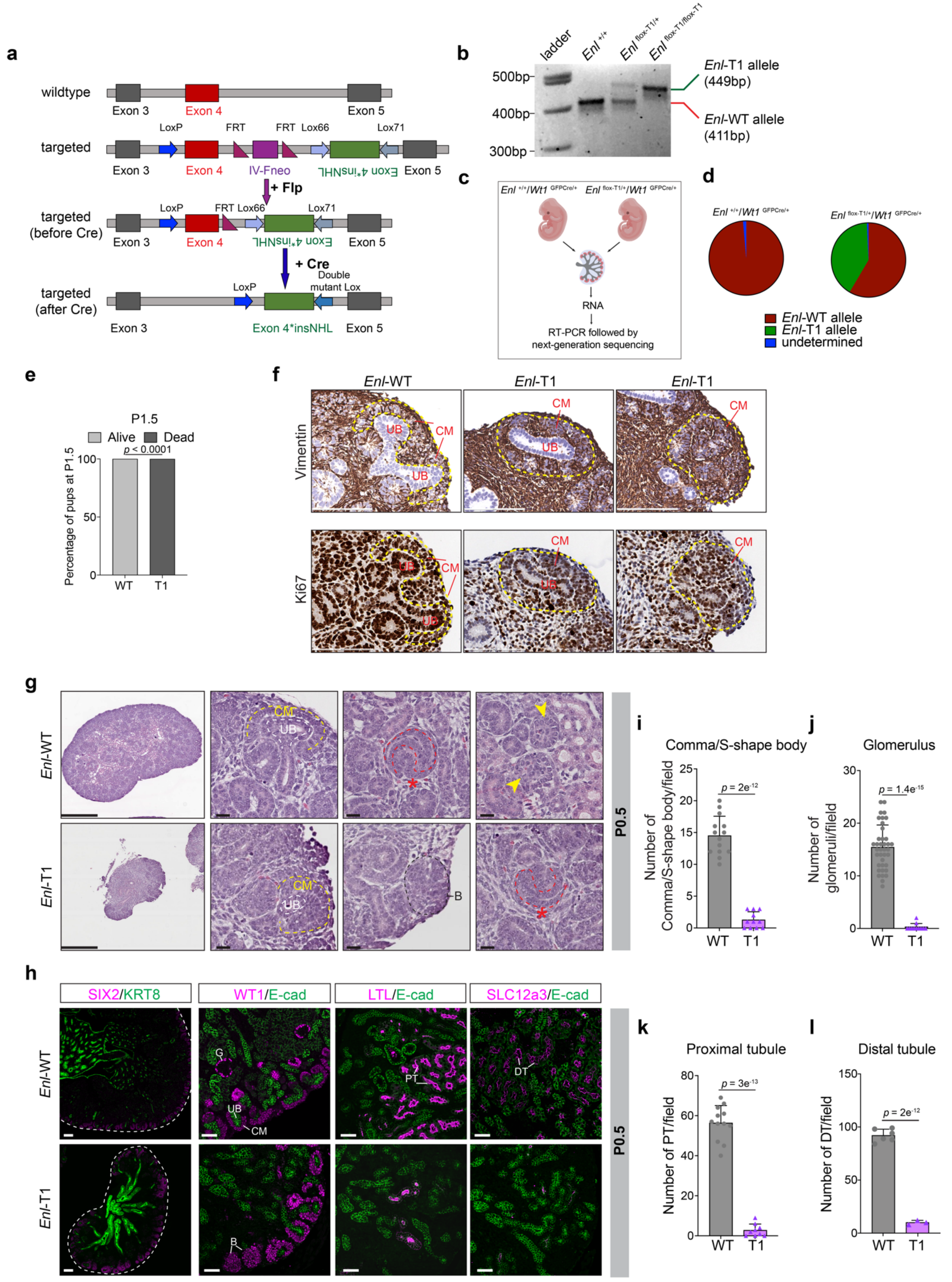


31

**Supplementary Figure 1. Heterozygous expression of mutant ENL disrupts embryonic kidney development and leads to postnatal mortality in *Wt1*$^{\mathbf{GFPCre/+}}$ mice.** **a**, Schematic of the *Enl*-T1 knock-in mouse model design. **b**, Gel image of genotyping PCR for indicated mice. **c**, Schematic of confirming the expression of *Enl*-T1 in kidney upon Cre-recombinase. **d**, The percentage of *Enl*-WT and *Enl*-T1 mRNA detected in E15.5 kidney. **e**, Quantification of survival status of *Enl*-WT and *Enl*-T1 pups at P1.5. **f**, Representive images of Ki-67 and Vimentin staining in E15.5 *Enl*-WT and *Enl*-T1 kidneys. Scale bar = 100 µm. **g**, Hematoxylin and eosin–stained sections showing the histology of P0.5 kidneys from *Enl*-WT and *Enl*-T1 pups. CM, cap mesenchyme; UB, ureteric bud. The red star indicates S-shape body, the black dashed line outlines a region of blastema-like structure (B), and the yellow arrows indicate glomerulus structures. Scale bar in the first column images, 500 µm; scale bar in the zoom-in images, 20 µm. **h**, Immunostaining for SIX2, KRT8, WT1, E-cadherin (E-cad), LTL, and SLC12a3 on P0.5 kidney sections. Scale bar in the first column images, 100 µm; scale bar in the zoom-in images, 50 µm. CM, cap mesenchyme; UB, ureteric bud; B, blastema-like structure; G, glomerulus; PT, proximal tubule; DT, distal tubule. **i**-**l**, Number of nephron structures per field. **i**, Comma/S-shape body ($n$ = 8 *Enl*-WT kidneys and 6 *Enl*-T1 kidneys); **j**, glomerulus ($n$ = 11 *Enl*-WT kidneys and 6 *Enl*-T1 kidneys); **k**, proximal tubule ($n$ = 5 *Enl*-WT kidneys and 6 *Enl*-T1 kidneys); **l**, distal tubule ($n$ = 4 *Enl*-WT kidneys and 4 *Enl*-T1 kidneys). One dot indicates the number of one indicated structure per field. Date represent mean ± s.d.; two-tailed unpaired Student's $t$-test. Source data are provided as a Source Data file.

## Supplementary Figure 2

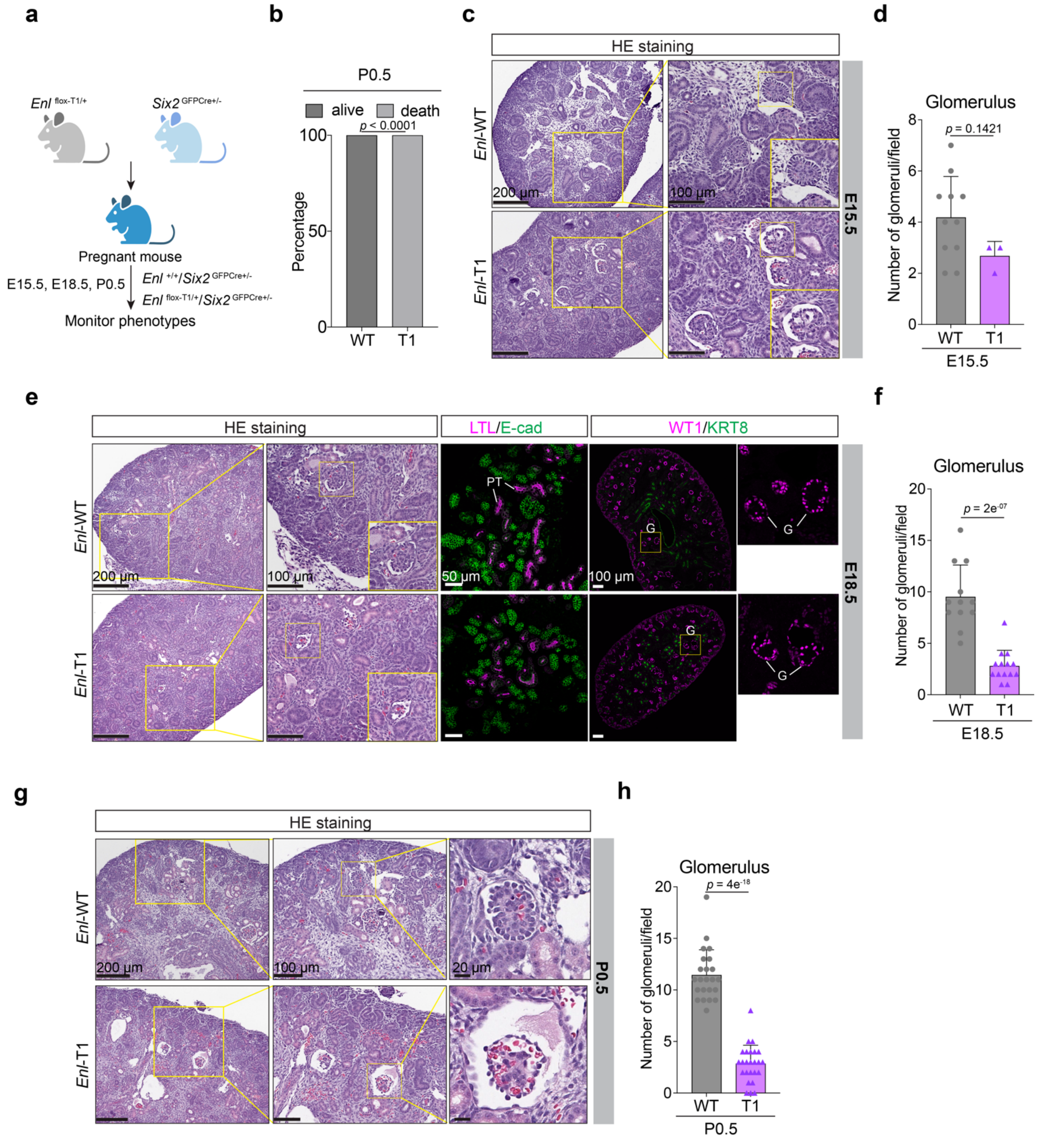

**Supplementary Figure 2. Heterozygous expression of mutant ENL disrupts embryonic kidney development and leads to postnatal mortality in *Six2*$^{GFPCre/+}$ mice.** **a**, Schematic of the breeding strategy. **b**, Quantification of survival status of *Enl*-WT and *Enl*-T1 pups at P0.5. **c**, **e**, **g**, Hematoxylin and eosin–stained sections showing the histology of E15.5 (**c**), E18.5 (**e**), and P0.5 (**g**) kidneys from *Enl*-WT and *Enl*-T1 pups. Scale bars are labeled in the images. **e**, Immunostaining for WT1, KRT8, E-cadherin (E-cad), and LTL on E18.5 kidney sections. Scale bars are labeled in the images. G, glomerulus; PT, proximal tubule. **d, f, h**, Number of glomeruli per field (E15.5, *n* = 6 *Enl*-WT kidneys and 2 *Enl*-T1 kidneys; E18.5, *n* = 4 *Enl*-WT kidneys and 6 *Enl*-T1 kidneys; P0.5, *n* = 8 *Enl*-WT kidneys and 9 *Enl*-T1 kidneys). One dot indicates the number of one indicated structure per field. Date represent mean ± s.d.; two-tailed unpaired Student's *t*-test. n.s., no significance. Source data are provided as a Source Data file.

## Supplementary Figure 3

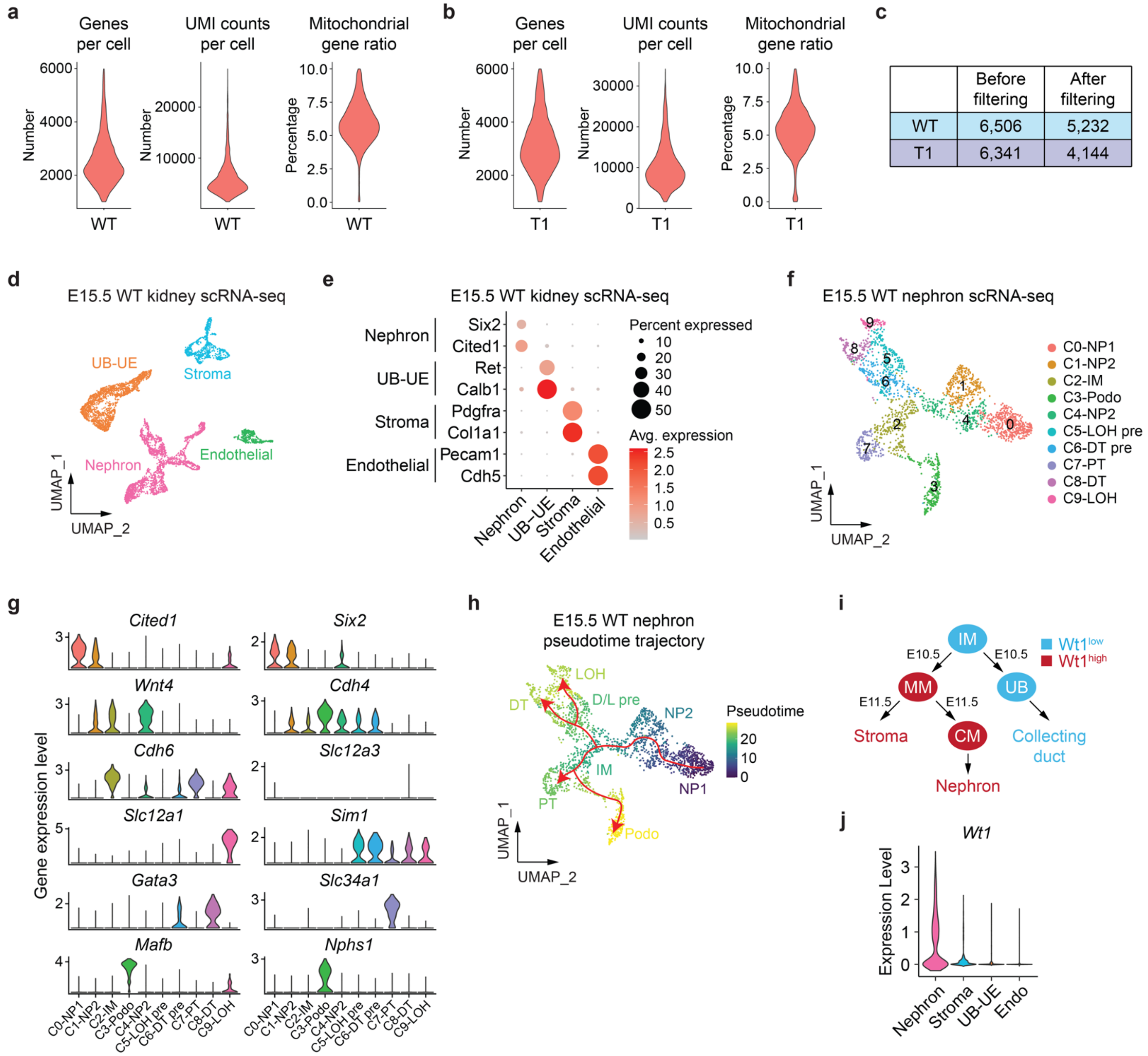


|  | Before filtering | After filtering |
|---|---|---|
| WT | 6,506 | 5,232 |
| T1 | 6,341 | 4,144 |

**Supplementary Figure 3. scRNA-seq identifies major cell types in *Enl*-WT embryonic kidneys. a**, **b**, Violin plot showing the number of informative genes per single cell, unique molecular identifiers (UMIs) per single cell, and mitochondrial gene ratio in scRNA-seq dataset of *Enl*-WT (**a**) and T1 (**b**) kidneys after quality control filtering. **c**, Table showing the number of cells before and after filtering according to the criteria written in Methods. **d**, UMAP embedding of *Enl*-WT kidney scRNA-seq data, with cells colored to represent the four main embryonic kidney lineages. UB, ureteric bud; UE, ureteric epithelium. **e**, Dot plot showing the expression of selected marker genes for each main kidney lineage. Color scale represents the average (Avg.) expression level. Circle size represents the percentage of cells expressing the gene. **f**, UMAP embedding of integrated scRNA-seq cells from *Enl*-WT nephrons. Cells are colored and labeled by annotated cell types. NP, nephron progenitor; IM, intermediate stage; Podo, podocyte; LOH pre, loop-of-Henle precursor; DT pre, distal tubule precursor; PT, proximal tubule; DT, distal tubule; LOH, loop-of-Henle. **g**, Violin plot showing the gene expression of selected makers for each cell type in *Enl*-WT nephrons. **h**, UMAP embedding of *Enl*-WT nephron scRNA-seq differentiation trajectory. Cells are colored by pseudotime. Trajectory is depicted by red arrows. **i**, Schematic illustrating the dynamics of *Wt1* gene expression in different cell lineages during nephrogenesis. Lowly and highly *Wt1* expressing lineages were colored in blue or red, respectively. IM, intermediate mesoderm; MM, metanephric mesenchyme. **j**, Violin plot showing the gene expression of *Wt1* in indicated cell lineages from *Enl*-WT kidneys. Endo, endothelial cells.

111 **Supplementary Figure 4**

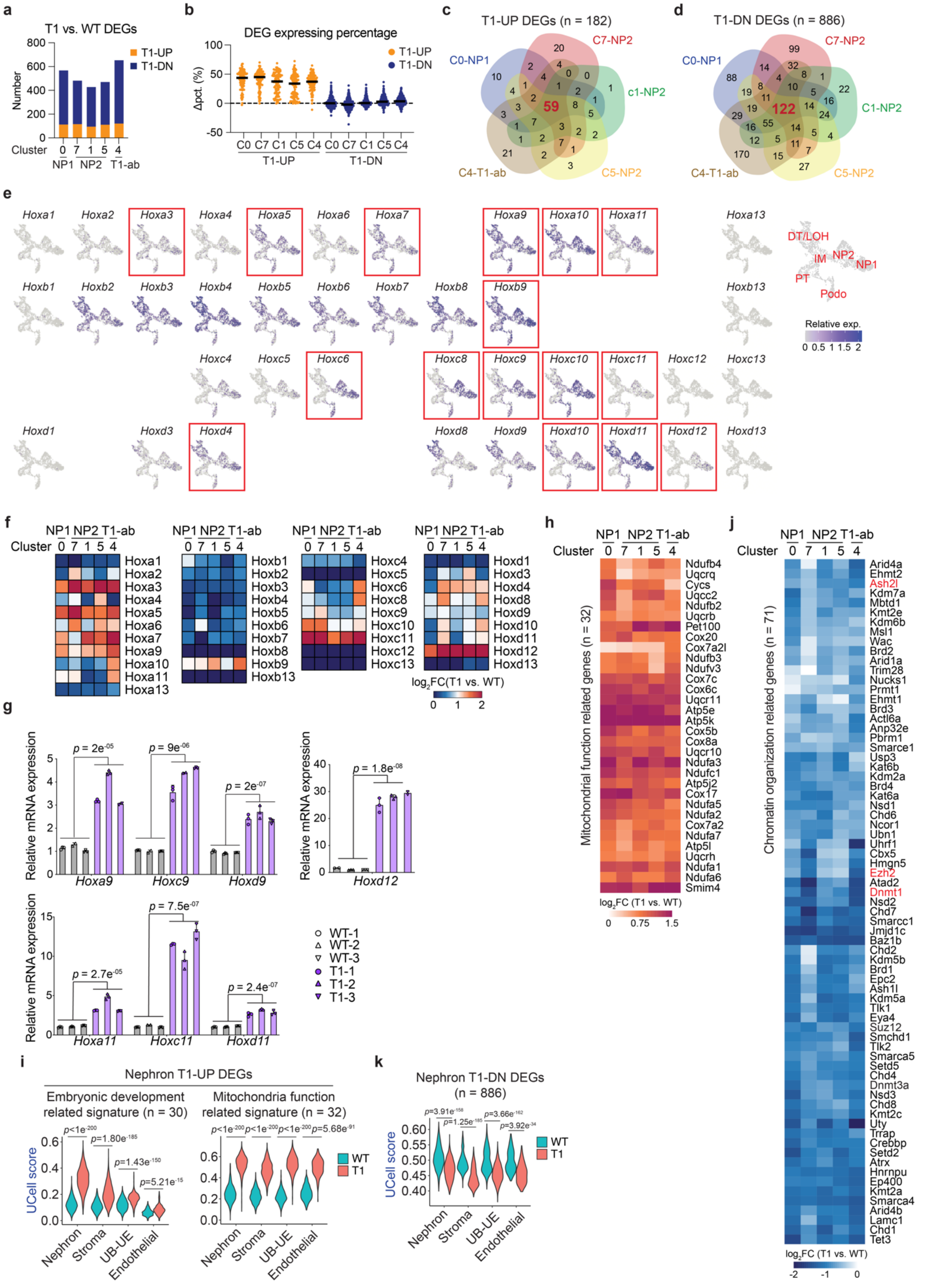


112

113

**Supplementary Figure 4. Transcriptional changes induced by mutant ENL in the developing kidney. a**, Stacked bar plot showing the number of DEGs between *Enl*-WT and T1 (upregulated in T1, T1-UP; downregulated in T1, T1-DN) in indicated nephron cell types. **b**, Dot plot showing the differential expressing percentage (Δpct.) of DEGs between *Enl*-WT and T1 in indicated nephron cell types. Δpct. = pct. (*Enl*-T1) - pct. (*Enl*-WT). T1-UP and DN DEGs were colored in orange or blue, respectively. **c**, **d**, Venn diagram showing the overlap among cell type specific T1-UP (**c**) or T1-DN (**d**) DEGs. **e**, UMAP embeddings showing all 39 *Hox* gene expression patterns in the *Enl*-WT nephron. Cells are colored by the expression level. The *Hox* genes that appeared in the union T1-UP DEG list were highlighted with a red box. Nephron cell types are labeled on the right. **f**, Heatmap showing the gene expression fold change (*Enl*-T1 versus WT) of all 39 *Hox* genes in indicated cell types. Fold change is scaled by log2. **g**, mRNA expression levels of *Hox* genes (normalized to *GAPDH*) in E15.5 *Enl*-WT and *Enl*-T1 kidneys. **h**, Heatmap showing the fold change of mitochondrial and metabolism-related T1-UP DEGs identified in **Figure 2i** within the indicated nephron cell types. Fold change is scaled by log2. **i**, **k**, Violin plot showing the UCell score evaluated by embryonic development-related (**i**, left), mitochondrial and metabolism-related (**i**, right), and union T1-DN (**k**) signatures for the four main embryonic kidney lineages within *Enl*-WT and T1 kidneys. Wilcoxon rank-sum test *p*-values are shown. **j**, Heatmap showing the fold change of chromatin organization-related T1-DN DEGs identified in **Figure 2m** within the indicated nephron cell types. Fold change is scaled by log2. Epigenetic regulators Dnmt1, Ezh2, and Ash2l are highlighted in red. Source data are provided as a Source Data file.

## Supplementary Figure 5

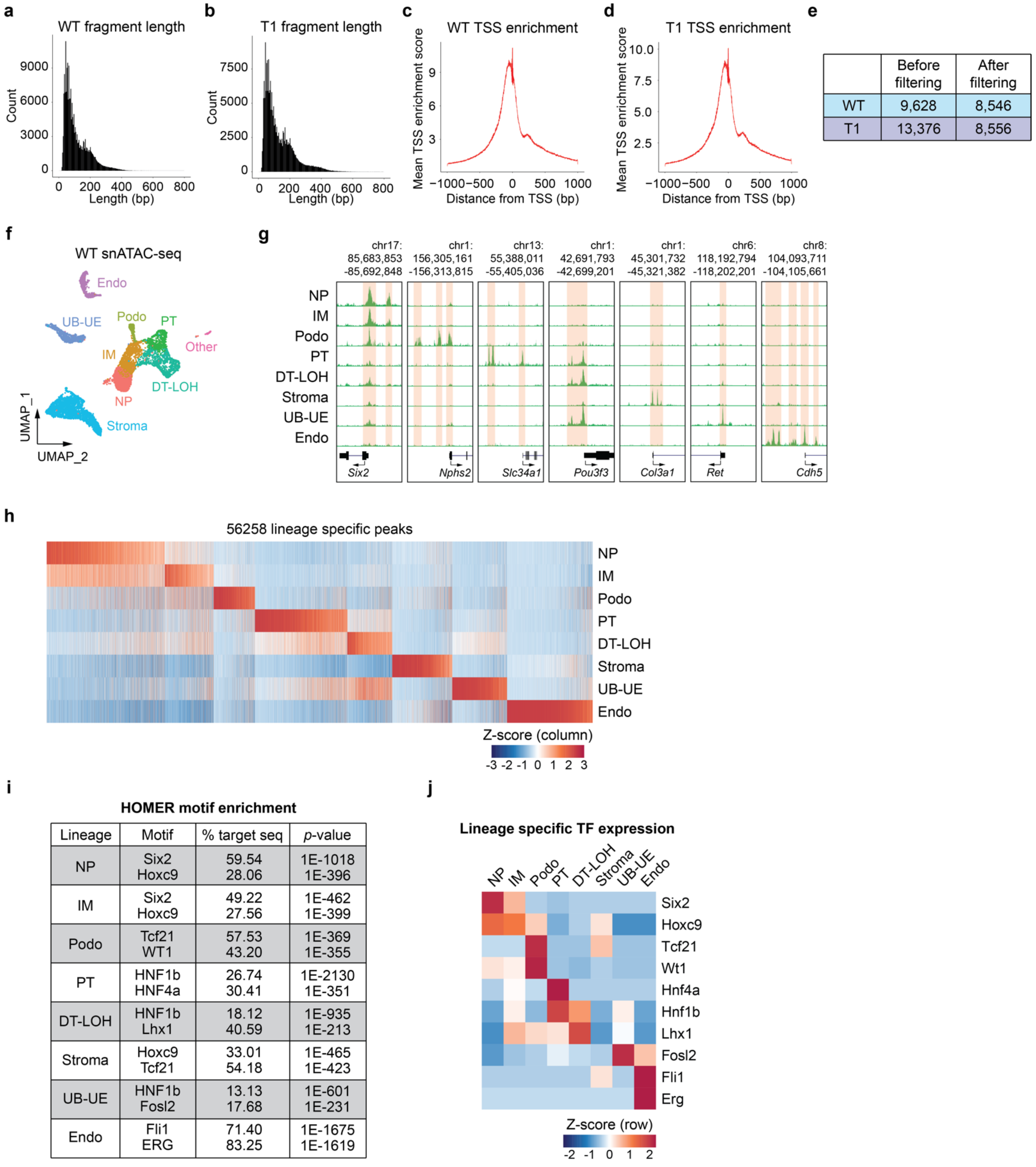


|  | Before filtering | After filtering |
|---|---|---|
| WT | 9,628 | 8,546 |
| T1 | 13,376 | 8,556 |

**HOMER motif enrichment**

| Lineage | Motif | % target seq | *p*-value |
|---|---|---|---|
| NP | Six2<br>Hoxc9 | 59.54<br>28.06 | 1E-1018<br>1E-396 |
| IM | Six2<br>Hoxc9 | 49.22<br>27.56 | 1E-462<br>1E-399 |
| Podo | Tcf21<br>WT1 | 57.53<br>43.20 | 1E-369<br>1E-355 |
| PT | HNF1b<br>HNF4a | 26.74<br>30.41 | 1E-2130<br>1E-351 |
| DT-LOH | HNF1b<br>Lhx1 | 18.12<br>40.59 | 1E-935<br>1E-213 |
| Stroma | Hoxc9<br>Tcf21 | 33.01<br>54.18 | 1E-465<br>1E-423 |
| UB-UE | HNF1b<br>Fosl2 | 13.13<br>17.68 | 1E-601<br>1E-231 |
| Endo | Fli1<br>ERG | 71.40<br>83.25 | 1E-1675<br>1E-1619 |

**Supplementary Figure 5. snATAC-seq reveals cell-type specific regulators in E15.5 *Enl*-WT kidney.** **a**, **b**, Insert size distribution of *Enl*-WT (**a**) and T1 (**b**) snATAC-seq data showing periodic patterns. **c**, **d**, Transcription start site (TSS) signal enrichment of the *Enl*-WT (**c**) and T1 (**d**) snATAC-seq data. **e**, Table showing the number of cells before or after filtering based on the criteria written in Methods. **f**, UMAP embedding of snATAC-seq cells from *Enl*-WT kidneys. Cells are colored by annotated cell types. **g**, Genome browser view of the ATAC signal in each snATAC-seq cell type annotated in (**f**) at selected marker gene promoters. **h**, Heatmap showing the relative ATAC signal of 56258 cell type/lineage-specific peaks among all cell types annotated in (**f**). See Supplementary Data 5. ATAC signals were normalized by column Z-score. **i**, Table showing the top 2 most significant TF candidates from the motif analysis for cell type-specific peaks identified in (**h**). **j**, Heatmap showing the expression level of the TF candidates listed in (**h**) in the corresponding *Enl*-WT scRNA-seq dataset. The gene expression was normalized by row Z-score.

## Supplementary Figure 6

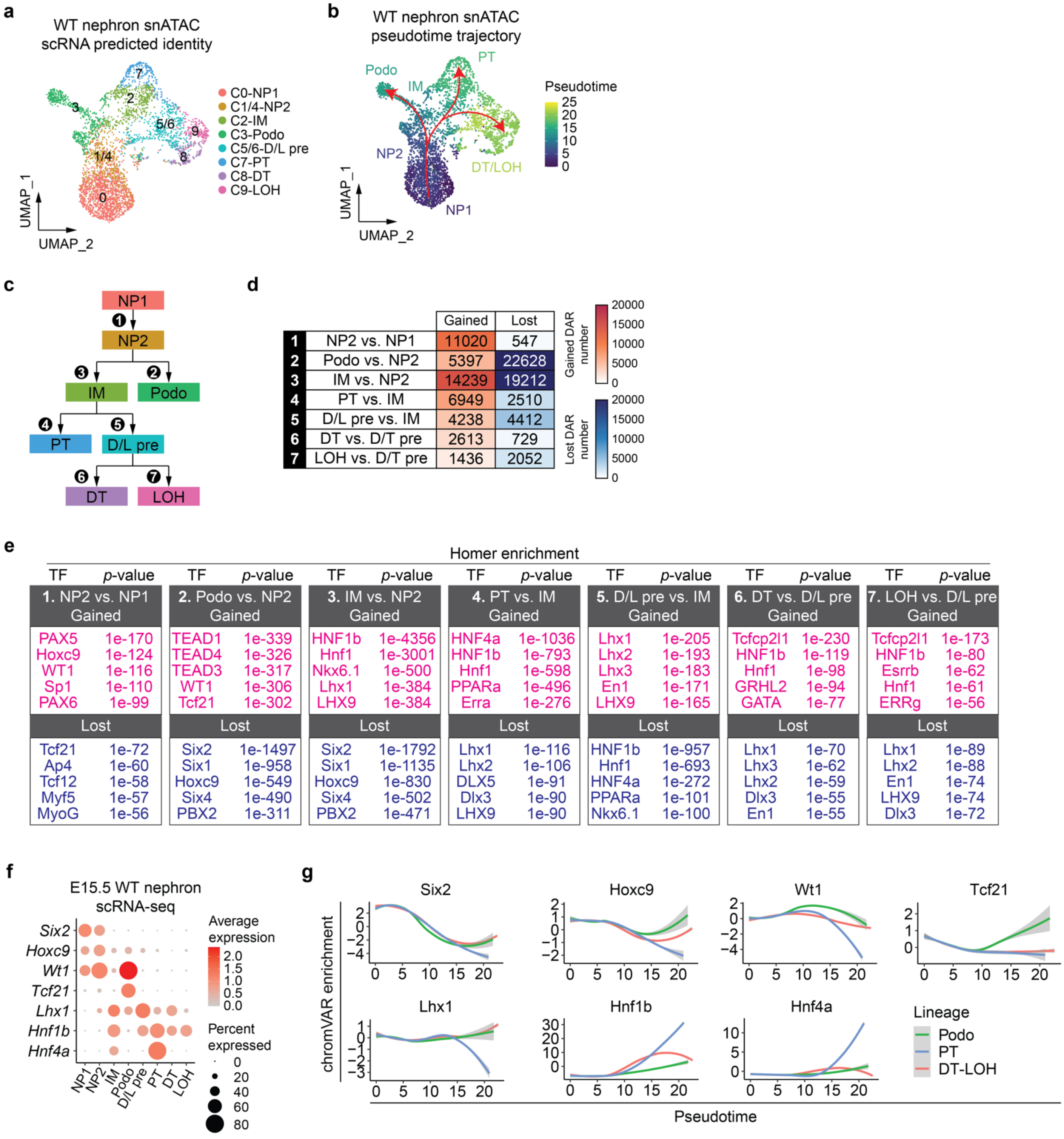

**Supplementary Figure 6. snATAC-seq reveals chromatin accessibility dynamics and the regulatory landscape during early nephrogenesis.** **a**, UMAP embedding of snATAC-seq cells from *Enl*-WT nephrons. Cells are colored and labeled by the cell types predicted by corresponding scRNA-seq data. **b**, UMAP embedding of *Enl*-WT snATAC-seq nephron differentiation trajectory. Cells are colored by pseudotime. Trajectories are depicted by red arrows. **c**, Diagram representing cell type divergence of *Enl*-WT nephrons. **d**, Table showing the number of differentially accessible regions (DARs) between subsequent stages in nephrogenesis. Cells were colored by the DAR number. Gained DARs were colored in red, lost DARs were colored in blue. See Supplementary Data 6. **e**, Table listing the top 5 most significant TF candidates from the motif analyses for the DARs identified in (**d**). TF candidates for gained and lost DARs were colored in red or blue, respectively. **f**, Dot plot showing the expression of selected TF candidates from (**e**) for each nephron cell type. Color scale represents the average expression level. Circle size represents the percentage of cells expressing the gene. **g**, Pseudotime-dependent dynamics of chromVAR TF enrichment score along Podo (green), PT (blue), and DT/LOH (red) differentiating trajectories.

## Supplementary Figure 7

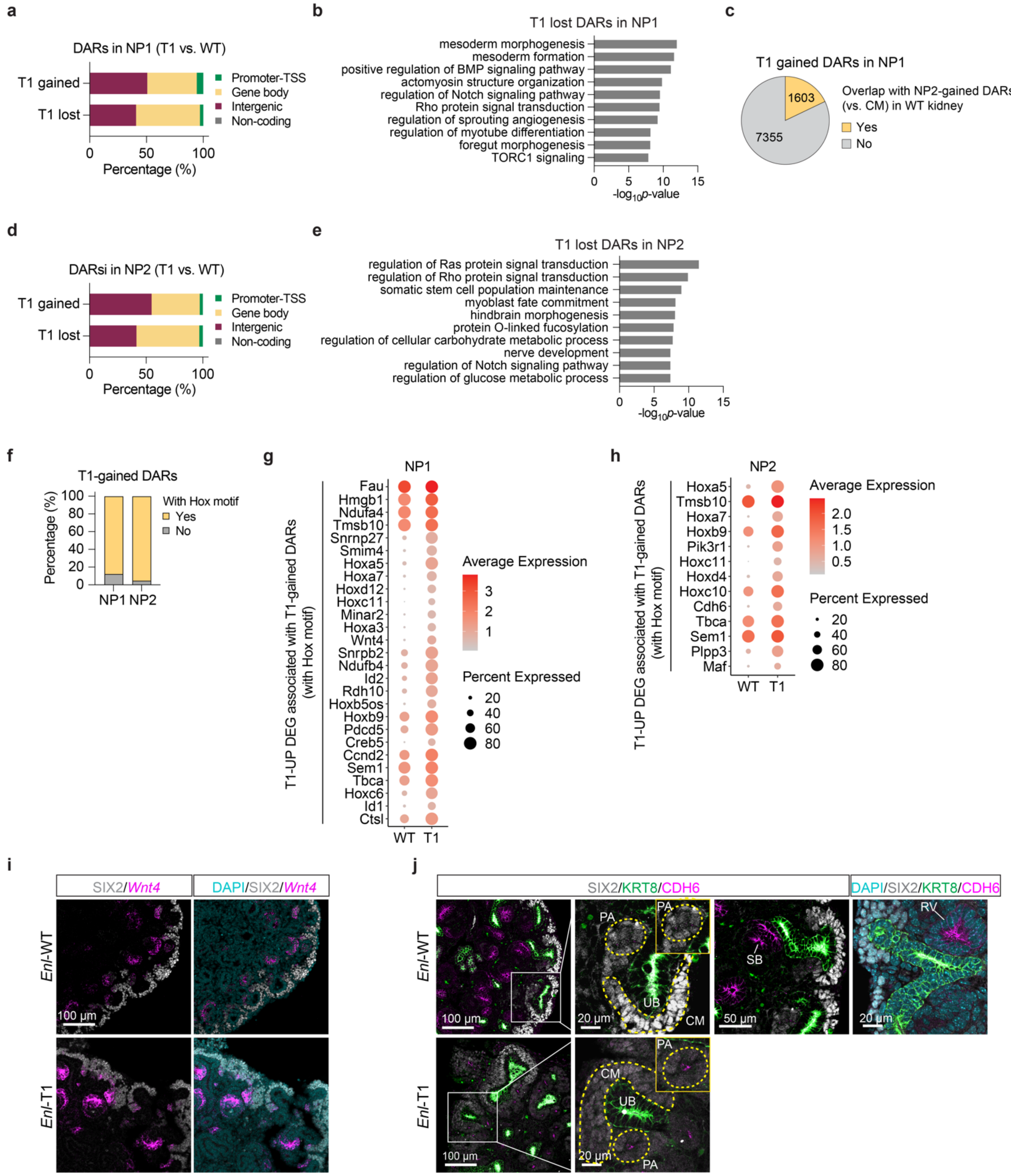

**Supplementary Figure 7. Mutant ENL promotes premature commitment of nephron progenitors while restricting their differentiation through misregulation of specific TF regulons.** **a**, **d**, Stacked bar plot showing the percentage of DARs located in promoter-TSS, gene body, intergenic, or non-coding regions. DARs were identified in NP1 (**a**) or NP2 (**d**) between *Enl*-WT and T1 cells. **b**, **e**, GREAT analysis of T1-lost DARs in NP1 (**b**) and NP2 (**e**). **c**, Pie chart showing the number of T1-gained DARs in NP1 overlapping with NP2-gained DARs (versus NP1) identified in *Enl*-WT kidneys. See Supplementary Data 8. **f**, Stacked bar plot showing the percentage of T1-gained DARs enriched with Hox TF motifs in NP1 (left) and NP2 (right). **g**, **h**, Dot plots showing the gene expression of Hox motif enriched T1-gained DARs associated T1-UP DEGs in NP1 (**g**) or NP2 (**h**). Color scale represents the average expression level. Circle size represents the percentage of cells expressing the gene. **i**, Representative images of SIX2/*Wnt4* mRNA co-staining in E15.5 kidneys. **j**, Representative images of SIX2/KRT8/CDH6 co-staining in E15.5 kidneys. CM, cap mesenchyme; UB, ureteric bud; PA, peritubular aggregate. SB, S-shape body. Data shown in **i** and **j** are representative of 3 *Enl*-WT kidneys and 3 *Enl*-T1 kidneys.

**Supplementary Figure 8**

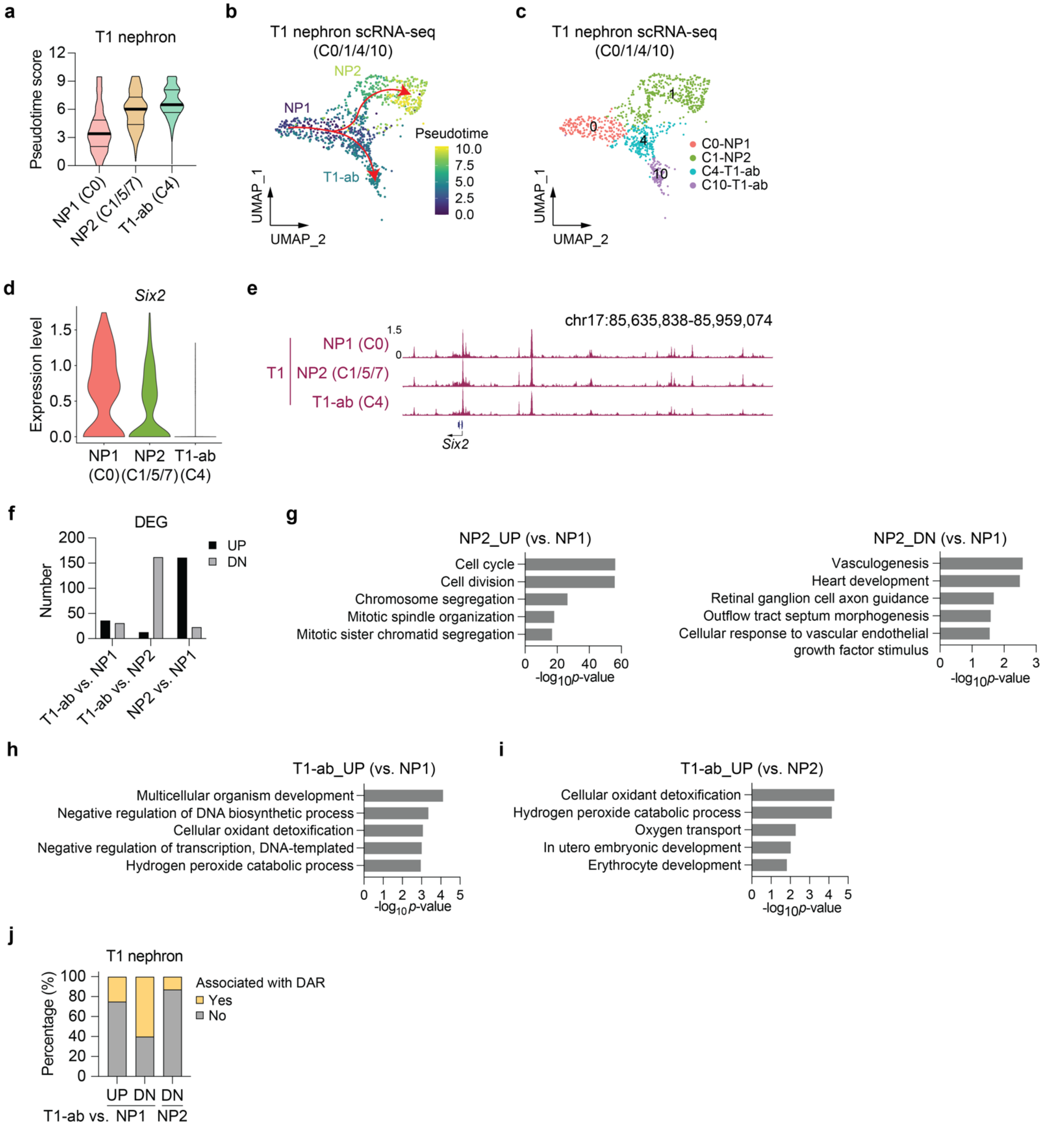

**Supplementary Figure 8. An abnormal progenitor state losing nephron chromatin identity emerges in the *Enl*-mutant kidney.** **a**, Violin plot showing pseudotime score of *Enl*-T1 cells clustered in NP1, NP2, and T1-ab. Thick center lines indicate median, top and bottom line limits are set to the 25th and 75th percentiles. **b**, UMAP embedding of scRNA-seq *ENL*-T1 nephron differentiation trajectory from NP1 to NP2 or T1-ab, colored by pseudotime score. Trajectories are depicted by red arrows. **c**, UMAP embeddings of *Enl*-T1 nephron (NP1, NP2, and T1-ab) scRNA-seq differentiation trajectory (**b**) and cell clustering (**c**). Cells were colored by pseudotime score (**b**) or cell type (**c**). **d**, Violin plot showing the expression level of *Six2* in indicated cell types from *Enl*-T1 nephrons. **e**, Genome browser view of ATAC signal at *Six2* locus in indicated cell types from *Enl*-T1 nephrons. **f**, Bar plot showing the number of DEGs identified in indicated comparison. Upregulated (UP) and down-regulated (DN) DEG numbers were colored in black or grey, respectively. See Supplementary Data 10. **g-i**, Bar plot showing the GO term analysis for the indicated DEGs. **j**, Stacked bar plot showing the percentage of DEGs associated with DARs identified in **Figure 5b** and **c**. See Supplementary Data 11.

# Supplementary Figure 9

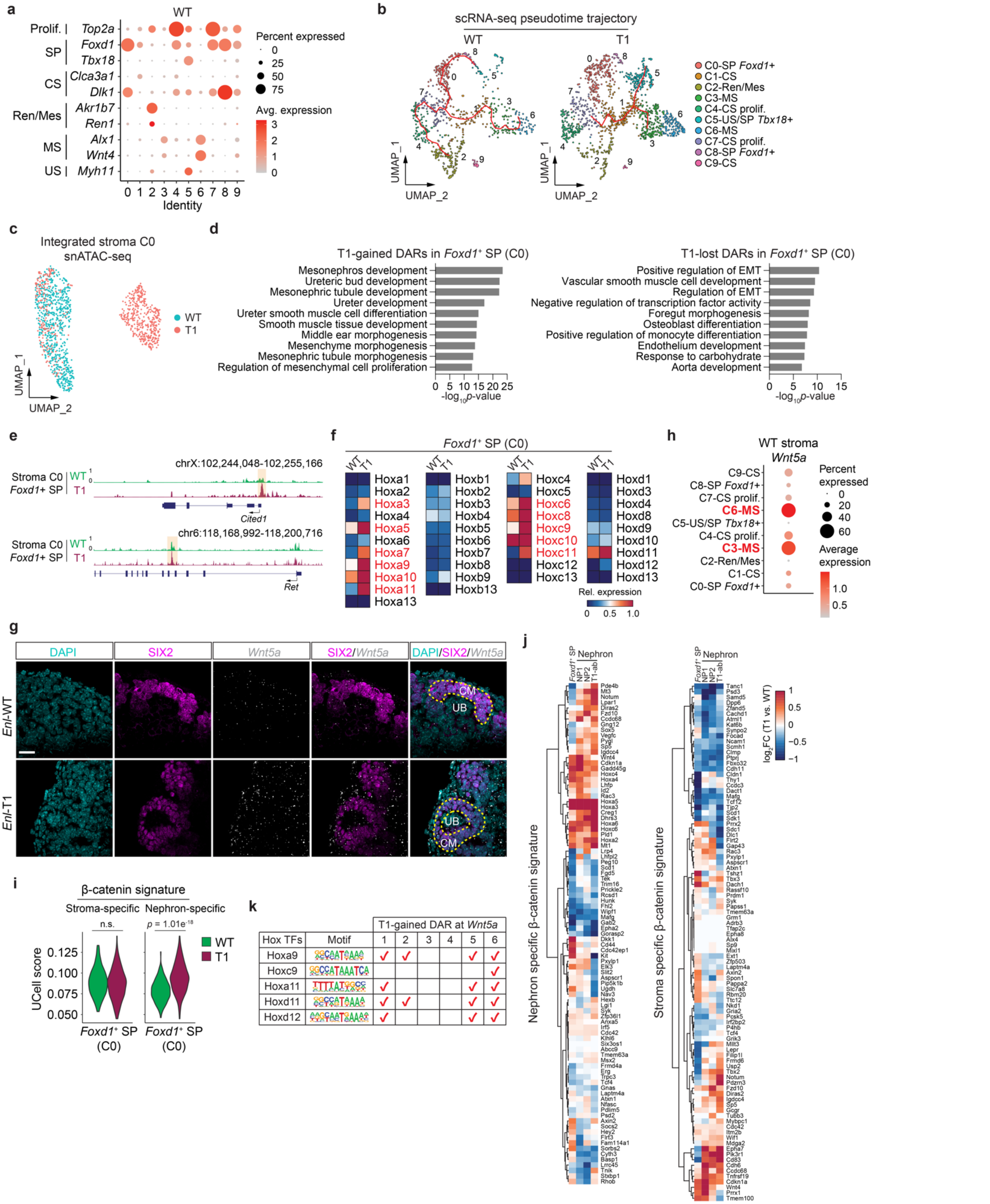

**Supplementary Figure 9. *Enl*-mutant *Foxd1*$^{+}$ stromal progenitors exhibit altered chromatin accessibility and might affect stroma-nephron interactions through aberrant activation of Wnt signaling. a**, The gene expression of cell type-specific marker genes in indicated cell clusters from *Enl*-WT stroma. Color scale represents the average expression level. Circle size represents the percentage of cells expressing the gene. SP, stromal progenitor; Prolif., proliferating cells; CS, cortical stroma; MS, medullary stroma; US, ureteric stroma; Ren/Mes, renin/mesangial cells; EMT, epithelial-mesenchymal transformation. **b**, UMAP embedding of *Enl*-WT (left) and T1 (right) scRNA-seq stroma differentiation trajectory. Cells were colored and labeled by cell types. Trajectories are depicted by red arrows. **c**, UMAP embedding of integrated snATAC-seq cells from *Enl*-WT and T1 stroma C0. Cells were colored by samples. **d**, GO term analyses for T1-gained (left) or lost (right) DARs in *Foxd1*$^{+}$ SP cells identified in **Figure 6f**. **e**, ATAC signal at *Cited1* (top) and *Ret* (bottom) gene loci in *Enl*-WT and T1 stroma C0 cells. **f**, The expression levels of all 39 *Hox* genes in *Enl*-WT and T1 stroma C0 cells. **g**, Representative images of SIX2/*Wnt5a* mRNA co-staining in E15.5 kidneys. Scale bar = 20 μm. Data shown in **g** have been done in 3 *Enl*-WT kidneys and 3 *Enl*-T1 kidneys with similar results. **h**, *Wnt5a* gene expression in each cell type of *Enl*-WT stroma. Color scale represents the average expression level. Circle size represents the percentage of cells expressing the gene. Two MS clusters with the highest *Wnt5a* expression level were highlighted in red. **i**, The UCell score evaluated by stroma (left) or nephron (right) specific β-catenin signature for cells from *Enl*-WT and T1 stroma. Wilcoxon rank-sum test *p*-values are shown. **j**, The fold change of expression level for the genes in nephron (left) or stroma (right) specific β-catenin signatures in indicated cell types. Fold change was scaled by log2. **k**, The enrichment of indicated Hox TF motifs within the T1-gained DARs in the *Wnt5a* gene locus.

## Supplementary Figure 10

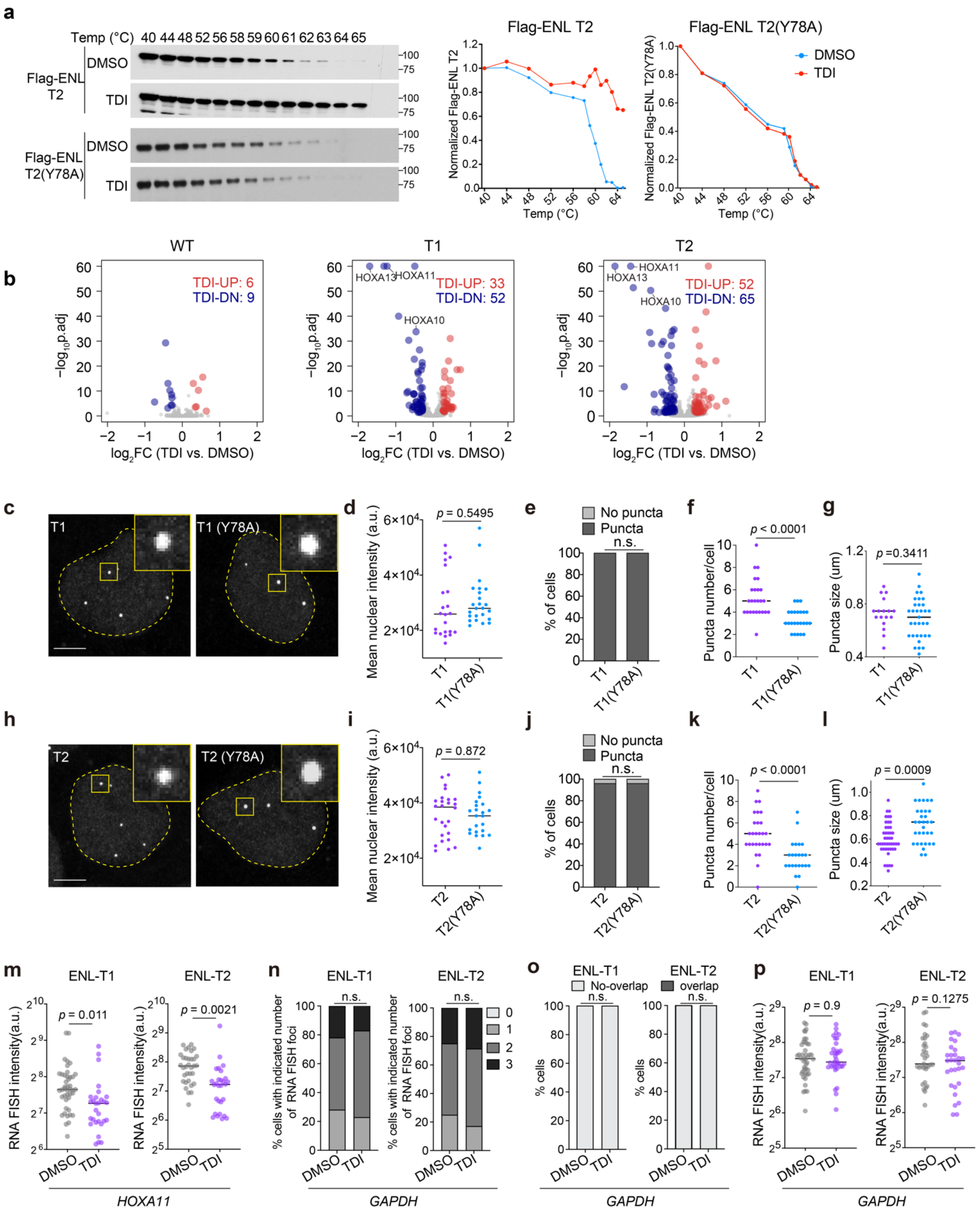

**Supplementary Figure 10. Blocking the acyl-binding activity of mutant ENL compromises its function on chromatin.** **a**, Immunoblots and quantification showing the levels of Flag-ENL T2 and Flag-ENL T2 (Y78A) after heat treatment in HEK293 cells at increasing temperatures. **b**, Volcano plot of RNA-seq data obtained from HEK293 cells expressing near endogenous levels of indicated transgenes with DMSO or TDI treatment. Data represent the mean across three replicates. See Supplementary Data 15. **c**-**k**, IF staining of Flag-ENL T1 and T1(Y78A) (**c**) and Flag-ENL T2 and T2(Y78A) (**h**) in HEK293 cells. Scale bar, 10 μm. Quantification of the mean nuclear intensity (**d**, $n$ = 22 T1 and 24 T1(Y78A); **i**, $n$ = 26 T2 and 23 T2(Y78A) ), the percentage of cells with or without condensates (**e**, **j**), condensate number in each nucleus (**f**, $n$ = 25 T1 and 24 T1(Y78A); **k**, $n$ = 27 T2 and 22 T2(Y78A) ) and condensate size (**g**, $n$ = 17 T1 and 33 T1(Y78A); **l**, $n$ = 44 T2 and 32 T2(Y78A)). **f**, **j**, **k**, **i**, Center lines indicate the median. **m**, Quantification of the mean intensity of *HOXA11* FISH foci ($n$ = 43 T1, 29 T1(Y78A), 33 T2, 28 T2(Y78A) ). Center lines indicate the median. **n**, Quantification showing the percentage of cells with the indicated number of *GAPDH* nascent RNA FISH foci (number indicates the number of RNA FISH foci in each nucleus). **o**, The percentage of cells containing *GAPDH* nascent RNA FISH foci overlapping with Flag-ENL condensates. Chi-square test; **p**, Quantification of the mean intensity of *GAPDH* FISH foci ($n$ = 43 T1, 35 T1(Y78A), 33 T2, 29 T2(Y78A)). Center lines indicate the median. **e**, **j**, Chi-square test, two-tailed. **f**, **g**, **k**, **i**, **m**, **p** Two-tailed unpaired Student's *t*-test. n.s., no significance. Source data are provided as a Source Data file.

## Supplementary Figure 11

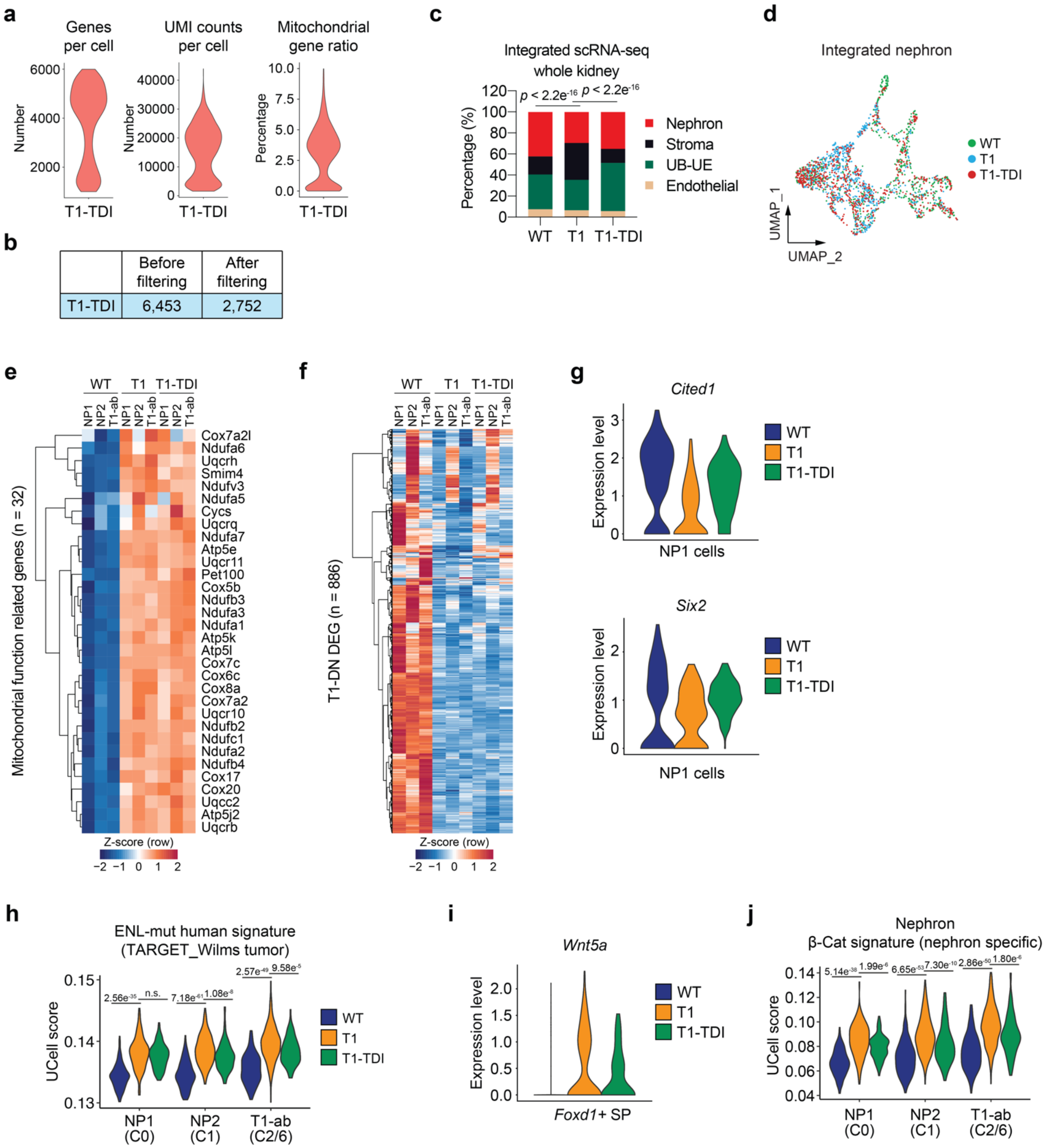

**Supplementary Figure 11. Transient treatment with TDI-11055 partially rescues mutant ENL-induced developmental and transcriptional defects in the developing kidney.** **a**, Violin plot showing the number of informative genes per single cell, unique molecular identifiers (UMIs) per single cell, and mitochondrial gene ratio in scRNA-seq dataset of *Enl*-T1 kidneys with TDI-11055 treatment (T1-TDI) after quality control filtering. **b**, Table showing the number of cells before and after filtering according to the criteria written in Methods. **c**, The percentage of four main embryonic kidney compartments within samples. Chi-Square test *p*-value is shown. **d**, UMAP embedding of integrated scRNA-seq data from *Enl*-WT/T1/T1-TDI nephrons. Cells were colored by sample. **e, f**, Heatmap showing the fold change of mitochondrial and metabolism-related T1-UP DEGs identified in **Figure 2i** (**e**) and union T1-DN DEGs identified in **Figure 2m (f)** within the indicated nephron cell types. Fold change is scaled by row z-score. **g**, Violin plot showing expression level of *Cited1* (top) and *Six2* (bottom) in NP1 from *Enl*-WT, T1, and T1-TDI nephrons. **h**, Violin plot showing the UCell score evaluated by human ENL-mut signature for the NP1, NP2, and T1-ab cell types from *Enl*-WT, T1, and T1-TDI nephrons. Wilcoxon rank-sum test *p*-values are shown. **i**, Violin plot showing expression level of *Wnt5a* in *Foxd1*$^{+}$ SP from *Enl*-WT, T1, and T1-TDI stroma. **j**, Violin plot showing the UCell score evaluated by nephron specific β-catenin signature for the NP1, NP2, and T1-ab cell types from *Enl*-WT, T1, and T1-TDI nephrons. Wilcoxon rank-sum test *p*-values are shown.

**Supplementary Figure 12**

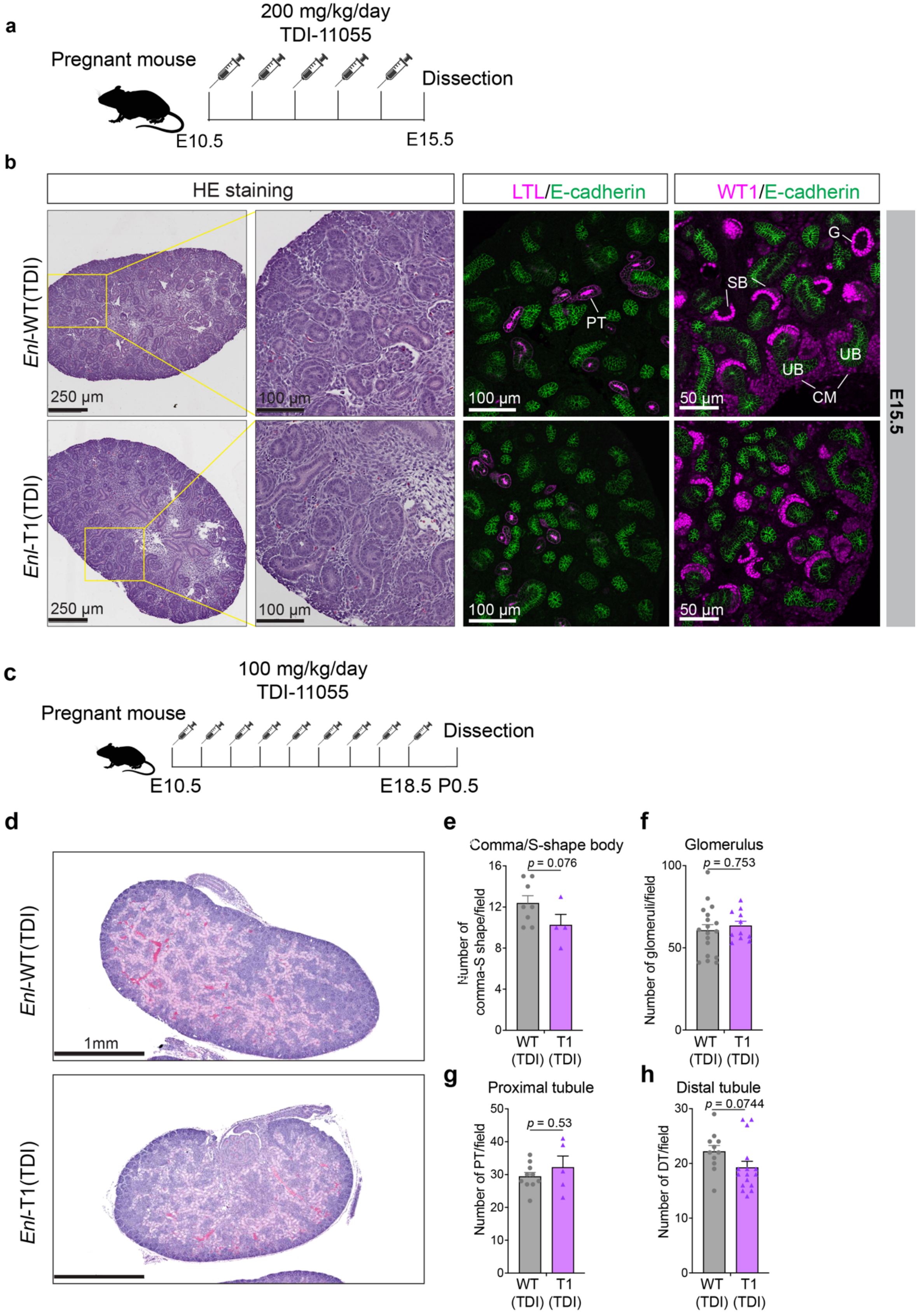

**Supplementary Figure 12. The impact of increasing the dosage of TDI-11055 or extending the treatment duration on rescuing mutant ENL-induced developmental defects in the kidney. a**, Schematic to show the experimental strategy. **b**, Histology and immunostaining for indicated proteins of E15.5 kidneys. CM, cap mesenchyme; UB, ureteric bud; SB, S-shape body; G, glomerulus; PT, proximal tubule. Images shown in b are representative of 4 *Enl*-WT(TDI) or 3 *Enl*-T1(TDI) kidneys. **c**, Schematic to show the experimental strategy. **d**, Histology of P0.5 kidneys. Scale bar, 1 mm. **e**-**h**, The number of nephron structures per field ($n$ = 4 for *Enl*-WT and *Enl*-T1 kidneys). One dot indicates the number of the indicated structure per field. Data represent mean ± s.d.; Two-tailed unpaired Student's *t*-test. Source data are provided as a Source Data file.

**Western blot images**

**Supplementary Figure 10a**

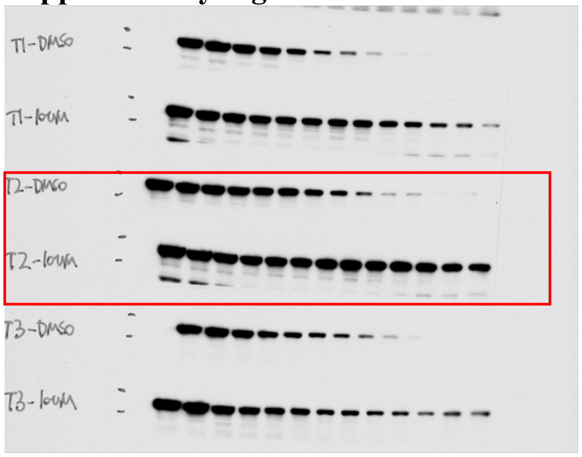


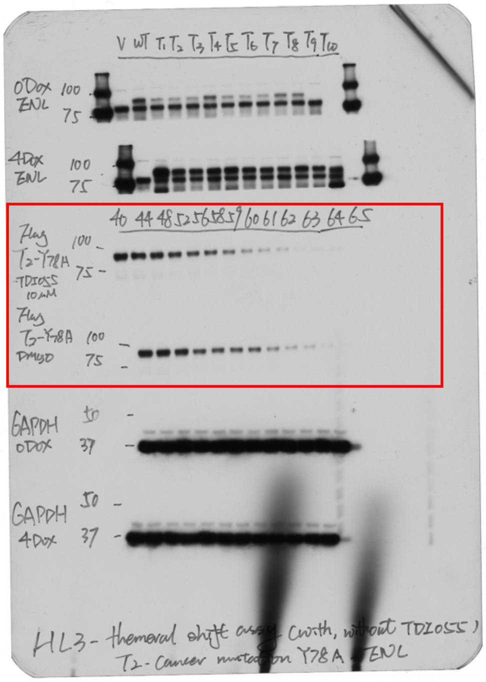

Supplement: Supplementary file 1 — Supplementary Information [file 41467_2024_50171_MOESM1_ESM.pdf]
