# Supplementary material for: Single-cell multiomics reveals ENL mutation perturbs kidney developmental trajectory by rewiring gene regulatory landscape
Source: Nat Commun. 2024 Jul 15;15:5937. doi: 10.1038/s41467-024-50171-w (PMC11250843; doi:10.1038/s41467-024-50171-w)
Supplement: Supplementary file 3 — Description of additional supplementary files [file 41467_2024_50171_MOESM3_ESM.pdf]

<u>Description of Additional Supplementary Files</u>

Supplementary data 1-Quality control metrics for all single-cell samples

Supplementary data 2-Genes differentially expressed between Enl-T1 and WT in nephron

Supplementary data 3-Human ENL-mut Wilms tumor signature

Supplementary data 4-TARGET-WT patients included in Fig. 2i and the corresponding GSVA scores of the human and mouse ENL-mut signatures

Supplementary data 5-Cell lineage specific ATAC peaks for Enl-WT kidney

Supplementary data 6-Differential accessibile regions during key cell type transitions in early nephrogenesis

Supplementary data 7-Differential accessibile regions between Enl-T1 and WT in integrated snATAC NP1/2 cells

Supplementary data 8-T1 gained DARs in NP1 which overlapped with NP2 gained DARs (vs. NP1) in EnlWT nephron

Supplementary data 9-Differential accessibile regions between T1-ab (C4) and NP1/2 in Enl-T1 snATAC nephron cells

Supplementary data 10-Genes differentially expressed among T1-ab (C4), NP1 and NP2 in Enl-T1 nephron cells

Supplementary data 11-Genes differentially expressed between T1-ab (C4) and NP1/2 associate with corresponding DARs in Enl-T1 nephron cells

Supplementary data 12-Differential accessibile regions between Enl-T1 and WT in integrated snATAC-seq stroma C0 cells

Supplementary data 13-28 stroma-nephron interaction related genes used in Fig. 6g

Supplementary data 14-Genes differentially expressed between DMSO and TDI-11055 treatment in ENLWT, T1 and T2 HEK293 cell lines

Supplementary data 15-Genes differentially expressed between ENL-T1/T2 and WT HEK293 cell lines

Supplementary data 16-Flag-ENL peaks in ENL-WT and T1 HEK293 cell lines under DMSO and TDI-11055 treatment

Supplementary data 17-T1 gained Flag-ENL peaks comparing with DMSO in ENL-T1 HEK293 cell line

Supplementary data 18-Primers for RT-qPCR
