# Supplementary material for: Single-cell multiomics reveals ENL mutation perturbs kidney developmental trajectory by rewiring gene regulatory landscape
Source: Nat Commun. 2024 Jul 15;15:5937. doi: 10.1038/s41467-024-50171-w (PMC11250843; doi:10.1038/s41467-024-50171-w)
Supplement: Supplementary file 5 — Reporting Summary [file 41467_2024_50171_MOESM5_ESM.pdf]

nature portfolio

Corresponding author(s): Liling Wan

Last updated by author(s): 2024/05/30

Reporting Summary

Nature Portfolio wishes to improve the reproducibility of the work that we publish. This form provides structure for consistency and transparency in reporting. For further information on Nature Portfolio policies, see our Editorial Policies and the Editorial Policy Checklist.

Statistics

For all statistical analyses, confirm that the following items are present in the figure legend, table legend, main text, or Methods section.

n/a	Confirmed	
☐	☒	The exact sample size (*n*) for each experimental group/condition, given as a discrete number and unit of measurement
☐	☒	A statement on whether measurements were taken from distinct samples or whether the same sample was measured repeatedly
☐	☒	The statistical test(s) used AND whether they are one- or two-sided<br>*Only common tests should be described solely by name; describe more complex techniques in the Methods section.*
☒	☐	A description of all covariates tested
☒	☐	A description of any assumptions or corrections, such as tests of normality and adjustment for multiple comparisons
☐	☒	A full description of the statistical parameters including central tendency (e.g. means) or other basic estimates (e.g. regression coefficient) AND variation (e.g. standard deviation) or associated estimates of uncertainty (e.g. confidence intervals)
☐	☒	For null hypothesis testing, the test statistic (e.g. *F*, *t*, *r*) with confidence intervals, effect sizes, degrees of freedom and *P* value noted<br>*Give P values as exact values whenever suitable.*
☒	☐	For Bayesian analysis, information on the choice of priors and Markov chain Monte Carlo settings
☒	☐	For hierarchical and complex designs, identification of the appropriate level for tests and full reporting of outcomes
☐	☒	Estimates of effect sizes (e.g. Cohen's *d*, Pearson's *r*), indicating how they were calculated

Our web collection on statistics for biologists contains articles on many of the points above.

Software and code

Policy information about availability of computer code

Data collection	Except for RNA FISH images, the other images were captured by LSM880 confocal microscope with 63x oil DIC objective in the Zeiss Zen Black software.<br>RNA-FISH images were captured by a widefield Leica microscope with 63x oil objective<br>Quantitative real-time PCR was perfomed with the ViiA 7 Real-time PCR System.<br>All sequencing samples generated in this study were prepared in house and sequenced on illumina platforms (Next-seq500/2000).
Data analysis	GraphPad Prism 10<br>Origin v.7.0<br>Image J<br>CellRanger v. 6.1.2<br>CellRanger ATAC v. 2.1.0<br>bedtools v. 2.26.0<br>Bowtie2 v. 2.2.5<br>CellPhoneDB v. 2.1.2<br>ChromVAR v. 3.1.0<br>Cicero v. 1.5.5<br>deeptools v. 3.5.2<br>DESeq2 v. 1.38.3<br>featureCounts v. 2.0.2<br>GenomicRanges v. 1.40.0<br>GREAT v. 4.0.4

GSEA v. 4.1.0
GSVA v3.17
HISAT2 v. 2.2.1
HOMER v. 4.10.4
MACS2 v. 2.2.8
Monocle3 v. 1.4.3
nichenetr v. 3.14
R v. 3.5.1
samtools v. 1.6
scATAC-pro v. 1.5.0
Seurat v. 4.3.0.1
SeuratWrappers v. 0.3.1
Signac v1.10.0
Sinto v0.9.0
snapATAC v. 1.0
UCell v. 2.4.0
UCSC bedgraphtobigwig v. 2.8
UCSC liftOver v.1.14.0

For manuscripts utilizing custom algorithms or software that are central to the research but not yet described in published literature, software must be made available to editors and reviewers. We strongly encourage code deposition in a community repository (e.g. GitHub). See the Nature Portfolio guidelines for submitting code & software for further information.

Data

Policy information about availability of data

All manuscripts must include a data availability statement. This statement should provide the following information, where applicable:

- Accession codes, unique identifiers, or web links for publicly available datasets
- A description of any restrictions on data availability
- For clinical datasets or third party data, please ensure that the statement adheres to our policy

Raw data, processed data, and metadata from mouse single-cell datasets have been deposited in GEO with the accession number GSE243868 and GSE243870. The ChIP-seq and RNA-seq data have been deposited in the Gene Expression Omnibus database under accession numbers GSE243866 and GSE243867. All other raw data generated or analyzed during this study are included in this published article (and its Supplementary Information files). Codes used for data analysis are available upon request.

Research involving human participants, their data, or biological material

Policy information about studies with human participants or human data. See also policy information about sex, gender (identity/presentation), and sexual orientation and race, ethnicity and racism.

Reporting on sex and gender	not applicable
Reporting on race, ethnicity, or other socially relevant groupings	not applicable
Population characteristics	not applicable
Recruitment	not applicable
Ethics oversight	not applicable

Note that full information on the approval of the study protocol must also be provided in the manuscript.

Field-specific reporting

Please select the one below that is the best fit for your research. If you are not sure, read the appropriate sections before making your selection.

☒ Life sciences ☐ Behavioural & social sciences ☐ Ecological, evolutionary & environmental sciences

For a reference copy of the document with all sections, see nature.com/documents/nr-reporting-summary-flat.pdf

Life sciences study design

All studies must disclose on these points even when the disclosure is negative.

Sample size	Sample size calculation was not conducted for this study. Sample size were determined according to standard practices. For the experiments (ITC assay, RT-qPCR, thermal shift assay, western blot, cellular immunofluorescence, and RNA-FISH), three independently biological replicates were used. RNA-seq involve three biological replicates. ChIP-seq experiment involved one replicate for each ell line. For in vivo experiments,

	we used at least 3 kidneys per group. The number of independent experiments and biological replicates was indicates in each figure legend.
Data exclusions	Sequencing data for scRNA-seq: For quality control, low-quality cells were excluded from further analysis based on the following criteria: (1) expressed gene number was < 1000 or > 6000 or (2) percentage of mitochondrial counts was > 10%.<br>Sequencing data for snATAC-seq: The cells without following the criterias were excluded for further analysis: (1) fragment number ranging from 3000 to 100,000, (2) percentage of fragments in peaks > 30%, (3) blacklist ratio < 2.5%, and (4) TSS enrichment score > 2.
Replication	The ITC assay, thermol shift assay, western blot, and RT-qPCR were conducted three times independently. The immunoflouscence for condensate formation and RNA-FISH were repeated three times. Three biological replicates for each group were involved in RNA-seq. ChIP-seq experiment involved one replicate for each cell line. All attempts at replication were successful.
Randomization	Randomization was not applicable to this manuscript since experimental mice were grouped by genotypes and the others are cell-culture based experiments.
Blinding	Blinding was not applicable. For both the wet lab experiments and NGS data analyses, we applied identical protocol and pipeline to individual samples, respectively.

Reporting for specific materials, systems and methods

We require information from authors about some types of materials, experimental systems and methods used in many studies. Here, indicate whether each material, system or method listed is relevant to your study. If you are not sure if a list item applies to your research, read the appropriate section before selecting a response.

Materials & experimental systems

n/a	Involved in the study
☐	☒ Antibodies
☐	☒ Eukaryotic cell lines
☒	☐ Palaeontology and archaeology
☐	☒ Animals and other organisms
☒	☐ Clinical data
☒	☐ Dual use research of concern
☒	☐ Plants

Methods

n/a	Involved in the study
☐	☒ ChIP-seq
☒	☐ Flow cytometry
☒	☐ MRI-based neuroimaging

Antibodies

Antibodies used	Antibody<br>Monoclonal ANTI-FLAG® M2 antibody Sigma-Aldrich Cat# F1804-1MG.Westernblot 1:1000; ChIP-seq, 6ug/ChIP; IF, 1:300.<br>Wt1 antibody Abcam Cat# Ab89901. IF, 1:50.<br>E-cadherin antibody Fisher Scientific Cat# BDB610181. IF, 1:400.<br>LTL antibody Vector laboratories Cat# B-1325. IF, 1:400.<br>Cadherin-16 antibody Santa Cruz Cat# sc-393132. IF, 1:50.<br>SLC12a3 antibody Abcam Cat# ab95302. IF, 1:100.<br>Six2 antibody Proteintech 1Cat# 1562-1-AP. IF, 100; ISH&IF, 1:50.<br>Cdh6 antibody Sigma-Aldrich Cat# HPA007047. IF, 1:50.<br>Anti-mouse IgG, HRP-linked Antibody Cell Signaling Technology Cat# 7074s. Western blot, 1:2000.<br>Anti-rabbit IgG, HRP-linked Antibody Cell Signaling Technology<br>Cat# 7076s. Western blot, 1:2000.<br>goat anti-Rabbit IgG (H+L) Alexa Fluor® 488 Invitrogen Cat# A32732. IF, 1:200.<br>goat anti-Mouse IgG (H+L) Alexa Fluor® 488 Invitrogen Cat# A32723. IF, 1:200.<br>goat anti-Rabbit IgG (H+L) Alexa Fluor® 568 Invitrogen Cat# A11011. IF, 1:200.<br>goat anti-Mouse IgG (H+L) Alexa Fluor® 568 Invitrogen Cat# A11031. IF, 1:200.<br>Streptavidin-Alexa Fluor™ 568 conjugate Thermofisher Cat# S11226. IF, 1:400.
Validation	The original scan of Western blot results were included with the Source Data file or Supplementary information file (*). Additionally, validation from the manufacturer was provided as a website link for reference.<br>Monoclonal ANTI-FLAG® M2 antibody Sigma-Aldrich Cat# F1804-1MG<br>https://www.sigmaaldrich.com/US/en/product/sigma/f1804<br>Wt1 antibody Abcam Cat# Ab89901<br>https://www.abcam.com/products/primary-antibodies/wilms-tumor-protein-antibody-can-r9ihc-56-2-ab89901.html<br>E-cadherin antibody Fisher Scientific Cat# BDB610181<br>https://www.bdbiosciences.com/en-us/products/reagents/microscopy-imaging-reagents/immunofluorescence-reagents/purified-mouse-anti-e-cadherin.610182<br>LTL antibody Vector laboratories Cat# B-1325<br>https://vectorlabs.com/products/biotinylated-lotus-tetragonolobus-lectin-ltl<br>Cadherin-16 antibody Santa Cruz Cat# sc-393132<br>https://www.scbt.com/p/cadherin-16-antibody-h-10?requestFrom=search

SLC12a3 antibody Abcam Cat# ab95302
https://www.abcam.com/products/primary-antibodies/slc12a3-antibody-ab95302.html
Six2 antibody Proteintech 11562-1-AP
https://www.ptglab.com/products/SIX2-Antibody-11562-1-AP.htm
Cdh6 antibody Sigma-Aldrich Cat# HPA007047
https://www.sigmaaldrich.com/US/en/product/sigma/HPA007047

Eukaryotic cell lines

Policy information about cell lines and Sex and Gender in Research

Cell line source(s)	HEK293 cell line was purchased from ATCC (ATCC CRL-1573). HEK293 lenti-teton-3xflag-ENL-WT/T1/T1(Y78A) and HEK293 lenti-teton-3xflag-Halo-ENL stable cells were generated in our lab.
Authentication	HEK293 cell line was tested for authentication by PCRC. STR profiling was used for authentication by PCRC.
Mycoplasma contamination	All cell line was mycoplasma-negative.
Commonly misidentified lines (See ICLAC register)	No misidentified lines were used in the study.

Animals and other research organisms

Policy information about studies involving animals; ARRIVE guidelines recommended for reporting animal research, and Sex and Gender in Research

Laboratory animals	The Wt1GFPCre/+ mouse strain (The Jackson Laboratory #010911): mice at 8-16-week-old were used for breeding; embryonic mice at E15.5 and E18.5 were used for control kidney collection; pups at P0.5 were used for control kidney collection.<br>The Six2GFPCre strain (kind gift from Susztak Lab): mice at 8-16-week-old were used for breeding; embryonic mice at E15.5 and E18.5 were used for control kidney collection; pups at P0.5 were used for control kidney collection.<br>The Enl-T1 knock-in mouse strain (Ingenious Targeting Laboratory): mice at 8-16-week-old were used for breeding.<br>The Enl flox-T1/+Wt1GFPCre/+ or Enl flox-T1/+Six2GFPCre/+ embryonic mice at E15.5 and E18.5 or pups at P0.5 were used for experimental kidney collection.<br>Mice were housed in a temperature-controlled specific-pathogen-free facility under 12 h light/dark cycles (lights on at 7:00 AM, off at 7:00 PM).
Wild animals	No wild animals were used.
Reporting on sex	Both male and female mice were used in the study.
Field-collected samples	No field-collected samples were used in the study.
Ethics oversight	All animal protocols are reviewed and approved by the Institutional Animal Care and Use Committees (IACUC) at the University of Pennsylvania.

Note that full information on the approval of the study protocol must also be provided in the manuscript.

Plants

Seed stocks	*Report on the source of all seed stocks or other plant material used. If applicable, state the seed stock centre and catalogue number. If plant specimens were collected from the field, describe the collection location, date and sampling procedures.*
Novel plant genotypes	*Describe the methods by which all novel plant genotypes were produced. This includes those generated by transgenic approaches, gene editing, chemical/radiation-based mutagenesis and hybridization. For transgenic lines, describe the transformation method, the number of independent lines analyzed and the generation upon which experiments were performed. For gene-edited lines, describe the editor used, the endogenous sequence targeted for editing, the targeting guide RNA sequence (if applicable) and how the editor was applied.*
Authentication	*Describe any authentication procedures for each seed stock used or novel genotype generated. Describe any experiments used to assess the effect of a mutation and, where applicable, how potential secondary effects (e.g. second site T-DNA insertions, mosiacism, off-target gene editing) were examined.*

ChIP-seq

Data deposition

☒ Confirm that both raw and final processed data have been deposited in a public database such as GEO.

☒ Confirm that you have deposited or provided access to graph files (e.g. BED files) for the called peaks.

Data access links<br>*May remain private before publication.*	https://www.ncbi.nlm.nih.gov/geo/query/acc.cgi?acc=GSE243866<br>Token: qlehmcekdlktbel

Files in database submission	HEK293 cells, Flag-ENL-WT, DMSO, ChIP-seq<br>HEK293 cells, Flag-ENL-WT, TDI-11055, ChIP-seq<br>HEK293 cells, Flag-ENL-T1, DMSO, ChIP-seq<br>HEK293 cells, Flag-ENL-T1, TDI-11055, ChIP-seq<br>HEK293 cells, Input, DMSO, ChIP-seq
Genome browser session (e.g. UCSC)	The corresponding BigWig files has been deposited to the GEO dataset above.

Methodology

Replicates	One replicate was involved in each group
Sequencing depth	Sample name, Total reads, Uniquely mapped reads, Sequencing strategy<br>HEK293_Flag-ENL-WT_DMSO,44195144,41238530,Single-end<br>HEK293_Flag-ENL-WT_TDI,38820656,36770636,Single-end<br>HEK293_Flag-ENL-T1_DMSO,32855336,30231020,Single-end<br>HEK293_Flag-ENL-T1_TDI,48461891,45880668,Single-end<br>HEK293_Input_DMSO,53835175,51803699,Single-end
Antibodies	Monoclonal ANTI-FLAG® M2 antibody Sigma-Aldrich Cat# F1804-1MG
Peak calling parameters	-f BAM -g hs --nomodel -p 1E-10 --broad --keep-dup all --broad-cutoff 1E-10
Data quality	MACS2 was used for Peak calling. The cut-off of p-value for peak calling is 1E-10. Input was used as control for peak calling.
Software	Bowtie2 (v2.2.5)<br>samtools (v1.6)<br>MACS2 (v2.2.8)<br>HOMER (v4.11)<br>Deeptools (v3.5.2)
